# Sensitivity of sea urchin fertilization to pH varies across a natural pH mosaic

**DOI:** 10.1002/ece3.2776

**Published:** 2017-02-12

**Authors:** Lydia Kapsenberg, Daniel K. Okamoto, Jessica M. Dutton, Gretchen E. Hofmann

**Affiliations:** ^1^ Department of Ecology Evolution and Marine BiologyUniversity of California Santa BarbaraSanta BarbaraCAUSA; ^2^ Sorbonne UniversitésUniversité Pierre et Marie Curie‐Paris 6CNRS‐INSULaboratoire d'Océanographie de VillefrancheVillefranche‐sur‐MerFrance; ^3^ School of Resource and Environmental ManagementSimon Fraser UniversityBurnabyBCCanada; ^4^ Wrigley Institute for Environmental StudiesUniversity of Southern CaliforniaLos AngelesCAUSA

**Keywords:** adaptive capacity, fertilization, ocean acidification, pH variability, *Strongylocentrotus*, sea urchin

## Abstract

In the coastal ocean, temporal fluctuations in pH vary dramatically across biogeographic ranges. How such spatial differences in pH variability regimes might shape ocean acidification resistance in marine species remains unknown. We assessed the pH sensitivity of the sea urchin *Strongylocentrotus purpuratus* in the context of ocean pH variability. Using unique male–female pairs, originating from three sites with similar mean pH but different variability and frequency of low pH (pH_T_ ≤ 7.8) exposures, fertilization was tested across a range of pH (pH_T_ 7.61–8.03) and sperm concentrations. High fertilization success was maintained at low pH via a slight right shift in the fertilization function across sperm concentration. This pH effect differed by site. Urchins from the site with the narrowest pH variability regime exhibited the greatest pH sensitivity. At this site, mechanistic fertilization dynamics models support a decrease in sperm–egg interaction rate with decreasing pH. The site differences in pH sensitivity build upon recent evidence of local pH adaptation in *S. purpuratus* and highlight the need to incorporate environmental variability in the study of global change biology.

## 1 Introduction

Species exist in spatially and temporally complex climatic environments. The role that such environmental complexity plays in shaping their sensitivity or resistance to anthropogenic climate change is not yet well understood for any biome (Boyd et al., 2016; Coble et al., 2016; Thornton, Ericksen, Herrero, & Challinor, 2014). According to the climate variability hypothesis, exposure to greater environmental variability leads to selection of wider climatic tolerance windows (Janzen, 1967; Stevens, 1989). This hypothesis is supported for temperature in both terrestrial and marine ectotherms (Sunday, Bates, & Dulvy, 2011). The close relationship between marine species’ temperature exposure and thermal tolerance is reflected by their distributions, which maximize the use of the species’ thermal niches (Sunday, Bates, & Dulvy, 2012). As such, ocean warming is expected to cause latitudinal shifts in species ranges (Sunday et al., 2012). The climate variability hypothesis provides a framework by which to study the importance of variability exposure in the assessment of species adaptive capacity (sensu Dawson, Jackson, House, Prentice, & Mace, 2011) to other environmental changes.

Compared to warming, predictive effects for other environmental changes, such as ocean acidification (i.e., decrease in seawater pH due to anthropogenic carbon dioxide, CO_2_, emissions) are trickier to determine in the absence of a strong latitudinal gradient (Kelly & Hofmann, 2013). Pending climate mitigation, ocean acidification is expected to yield a decrease in global mean seawater pH_T_ (pH on the total hydrogen ion scale) of 0.13–0.42 (Pörtner et al., 2014). Much like temperature, this mean change is dwarfed by natural variability, which on a global scale ranges between pH_T_ 7.8 and 8.4 (Rhein et al., 2013). Locally, ocean pH variability regimes arise due to geographic differences in oceanographic and biological features. Variation in these can create natural hot spots of (Hofmann et al., 2014), or refuges from (Kapsenberg & Hofmann, 2016), harmful low pH exposures (e.g., pH_T_ < 7.7). The potential selection pressure that pH variability envelopes impose is not well studied or understood.

**Figure 1:**
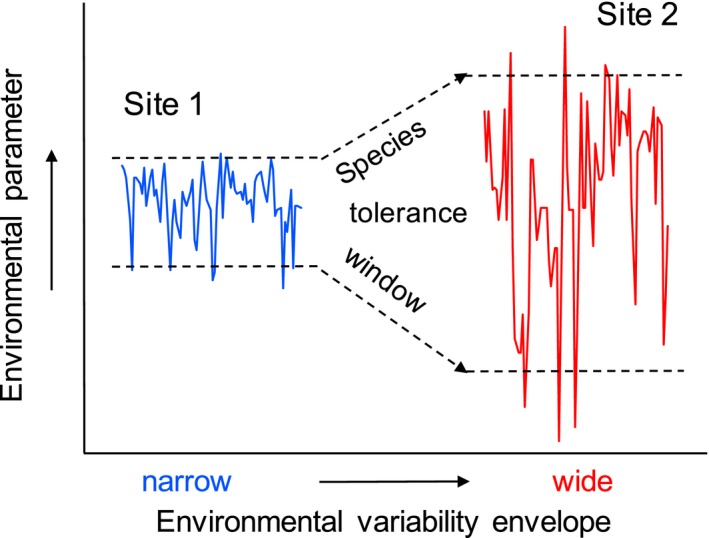
Conceptual diagram describing the climate variability hypothesis. In the context of ocean acidification, the environmental parameter is pH. As low pH exposures increase in frequency and the pH variability envelope widens (Site 2), the pH range within which organismal physiology must operate also widens. Through local environmental conditioning or natural selection, this may cause spatial differences in a species’ tolerance window across its biogeographic range (dashed lines)

Here, we investigate whether or not pH tolerance is related to local pH variability regimes (defined here as sites with high variance in pH time series observations and frequent exposure to low pH). As the pH variability range increases, the pH range within which organismal physiology must operate widens, creating the environmental regime that would select for fertilization kinetics that are resistant to low pH (Figure 1). Observing this effect in natural populations is extremely valuable as it infers transgenerational plasticity (via maternal provisioning or epigenetic modification Ross, Parker, & Byrne, 2016; Hofmann, 2017) or local adaptation and a potential means for genetic adaptation to future ocean acidification (Hofmann et al., 2014; Kelly & Hofmann, 2013; Kelly, Padilla‐Gamiño, & Hofmann, 2013; Pespeni, Chan, Menge, & Palumbi, 2013). Species that can adapt to spatial environmental differences may be better equipped to adapt to temporal changes, such as ocean acidification.

We investigated fertilization success in the purple sea urchin *Strongylocentrotus purpuratus* (Pearse, 2006), across urchin groups spanning radically different pH variability regimes in the eastern boundary California Current Large Marine Ecosystem (CCLME, NE Pacific Ocean). In the CCLME, periodic upwelling brings deep, cold, low‐pH (pH_T_ < 7.7) seawater to the coast (Feely, Sabine, Hernandez‐Ayon, Ianson, & Hales, 2008; Hofmann et al., 2011). While extreme low pH events (pH_T_ < 7.60) are rare, these conditions are likely to become more frequent as upwelling events have increased in strength and duration (Iles et al., 2012) and as ocean acidification progresses (Gruber et al., 2012). Due to spatial variability of upwelling intensity along the CCLME coastline (Feely et al., 2008), the magnitude and frequency of low pH exposures are spatially constrained. These locales encapsulate the biogeographic range of diverse benthic marine invertebrates.

Despite spanning a large biogeographic range from Alaska to Baja California and colonizing both subtidal and intertidal habitats, *S. purpuratus* lacks a strong genetic structure (Edmands, Moberg, & Burton, 1996; Flowers, Schroeter, & Burton, 2002; Palumbi & Wilson, 1990; Pespeni, Oliver, Manier, & Palumbi, 2010). As such, larvae exhibit little to no differences in temperature sensitivity across sites spanning a ~10°C gradient (Hammond & Hofmann, 2010). Yet, recent studies show that *S. purpuratus* is influenced by in situ pH exposures where the resistance to ocean acidification is mediated in part by natural selection through differential pH exposures (as shown for larval growth, Kelly et al., 2013; and genomewide allelic frequencies, Pespeni, Chan, et al., 2013). *S. purpuratus* is a broadcast spawner, and thus, fertilization is exposed directly to seawater pH. Given that processes which regulate fertilization are frequently under strong selective pressures (Levitan, 2002, 2004), pH sensitivity in fertilization dynamics has the potential to introduce strong selective pressure for pH tolerance.

Previous studies of urchin fertilization in the context of ocean acidification have revealed a range of pH sensitivities. Responses include reduced fertilization success (Kurihara & Shirayama, 2004; Moulin, Catarino, Claessens, & Dubois, 2011; Reuter, Lotterhos, Crim, Thompson, & Harley, 2011) and sperm motility (Campbell, Levitan, Hosken, & Lewis, 2016; Havenhand, Buttler, Thorndyke, & Williamson, 2008; Schlegel, Havenhand, Gillings, & Williamson, 2012); however, there are some contradictory observations (Byrne, Soars, Selvakumaraswamy, Dworjanyn, & Davis, 2010; Caldwell et al., 2011). In addition, species differences (Frieder, 2014), pair‐specific pH sensitivities (Schlegel et al., 2012; Sewell, Millar, Yu, Kapsenberg, & Hofmann, 2014), and sensitivity that scales with tide pool exposure (based on 2 days of observations, Moulin et al., 2011) have been reported. In addition, individuals within populations may be differentially impacted by low pH (Campbell et al., 2016). There is a need to expand fertilization experiments across species’ biogeographic ranges and incorporate in situ exposures to the interpretation of results (Havenhand & Schlegel, 2009; Moulin et al., 2011). Using urchins from three sites spanning 1,500 km and different in situ pH exposures, we exposed eggs to a gradient of sperm concentrations under various pH levels to assess fertilization in the laboratory. As *S. purpuratus* lacks a strong genetic structure across these sites, site differences in fertilization success would be attributable to local environmental effects on adult condition or gamete quality.

## 2 Materials and Methods

### 2.1 Sites and field pH exposures

**Figure 2:**
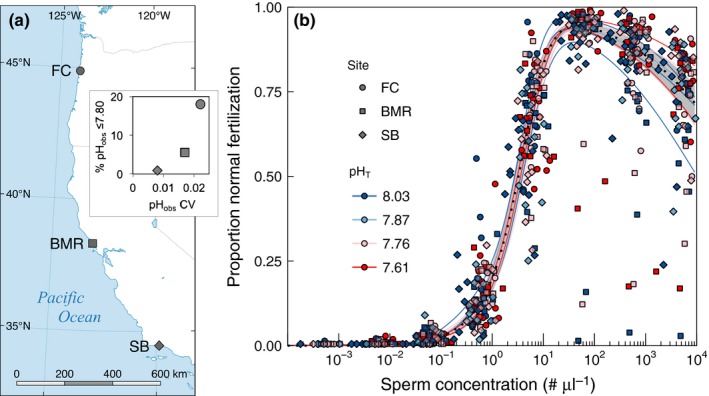
Map of study sites in the California Current Large Marine Ecosystem (a) and normal fertilization (NF) functions of *Strongylocentrotus purpuratus* from those sites by pH treatment (b). Sites span >10° latitude from Oregon (FC) to California (BMR, SB) and exhibit similar mean pH values but unique pH variability regimes as described by percent of pH observations ≤7.80 and coefficient of variation (CV) of pH sensor observations (FC and BMR, April‐October, 2011–2013; SB, 2012–2015). For the NF functions (b), the gray band with dotted line represents the “global” normal fertilization curve (pooled site and pH treatment) at the 95% confidence interval, while points and thin lines represent mean site–pH treatment combinations. Extreme outliers under the fertilization curve originate largely from two pairs from BMR

Three sites with distinct pH variability regimes were chosen (Figure 2a): Fogarty Creek, Oregon (FC, 44°50.200N, 124°03.517W, intertidal), Bodega Marine Reserve, California (BMR, 38°19.110N, 123°04.452W, intertidal), and Goleta Pier in the Santa Barbara Channel, California (SB, 34°24.854N, 119°49.711W, subtidal). Field pH exposures at each site were measured by autonomous, custom‐built, Honeywell Durafet^®^‐based pH sensors. These were deployed in the intertidal zone on emergent rocky benches during the upwelling season at FC and BMR, from April to October in 2011–2013. A detailed oceanographic description of pH exposures across the CCLME, including FC and BMR, is forthcoming (Chan et al., in review). For SB, SeaFET pH sensors (Martz, Connery, & Johnson, 2010) were deployed on a subtidal mooring from 2012 to 2015 (Mohawk Reef, 34°23.66N, 119°43.80W), <10 km from the SB urchin collection site (Hofmann & Washburn, 2015).

Mean pH_T_ conditions of the time series at each site were similar (pH_T_ 7.99–8.01), but variability and frequency of low pH exposures differed (Figure 2a). FC experiences strong, seasonal upwelling events resulting in the most frequent exposures to low pH (18.1% of pH_T_ observations were ≤7.80). BMR experiences more intermediate upwelling strength resulting in less frequent low pH exposure and less overall pH variability (5.6% of pH_T_ observations were ≤7.80), compared to FC. SB experiences the weakest upwelling and narrowest pH variability regime with <1% of pH_T_ observations ≤7.80. As the study period at FC and BMR targeted the upwelling season, frequency of low pH_T_ (≤7.80) exposures is likely to be lower over an annual period than that reported here but still expected to be greater than at SB.

### 2.2 Seawater treatments

For fertilization trials, we confined treatment conditions (three per site) to the range of pH observations at the study sites, rationalized by the assumptions that (1) present‐day rare pH exposures will become more frequent in the future, and (2) species may not have been exposed to more extreme values than recently observed. Treatments for SB urchins were pH_T_ 8.03, 7.87, and 7.76, whereas FC and BMR treatments were pH_T_ 8.03, 7.76, and 7.61. Seawater acidification was performed by bubbling mixed dry, CO_2_‐free air and pure CO_2_ gas at desired pCO_2_ levels via venturi injectors in sealed reservoir buckets filled with 0.35 μm filtered, UV‐sterilized seawater (FSW) and maintained in temperature‐controlled sea tables at ~15°C (Aqua Logic Inc., Digital temperature controller), following modified methods from Fangue et al. (2010).

In order to isolate pH effects, temperature was held constant. The spawning environment of *S. purpuratus* is not homogenous at 15°C and low pH often occurs at low temperatures (Hofmann et al., 2014; Reum et al., 2016). As such, the pH treatments intentionally do not fully represent future habitat exposures. Notably, marine invertebrate fertilization is generally tolerant across a wide range of temperatures (Byrne, 2011).

For each fertilization trial, treatment water was sampled from the reservoirs for salinity (YSI 3100 Conductivity Instrument) and total alkalinity (A_T_, open‐cell titration using Mettler‐Toledo T50 titrator) following standard operating procedures (Dickson, Sabine, & Christian, 2007). Temperature and pH were measured immediately at the end of the fertilization trials from control vials that were handled experimentally without sperm or egg additions. pH_T25°C_ was determined spectrophotometrically (Dickson et al., 2007) using unpurified m‐cresol purple (Sigma‐Aldrich^®^). This method potentially underestimates pH_T_ by 0.032 ± 0.006 (Kapsenberg, Kelley, Shaw, Martz, & Hofmann, 2015). Carbonate system parameters were calculated at in situ temperatures from pH_T25°C_, A_T_, and salinity using the program CO2Calc [Version 1.0.1, 2010, U.S. Geological Survey] with dissociation constants from Mehrbach, Culberso, Hawley, and Pytkowic (1973) refit by Dickson and Millero (1987).

### 2.3 Animal collections and fertilization trials

Adult *S. purpuratus* were collected in 2011 at a subtidal rocky reef at SB in February and from emergent rocky benches and surge channels in the intertidal at BMR and FC in March and April, respectively (Figure 2a). Sampling from tide pools was avoided as this would have introduced undocumented pH exposures that may influence fertilization (Kwiatkowski et al., 2016; Moulin et al., 2011). Collection location (subtidal vs. low intertidal) effects on fertilization have not previously been detected in an echinoderm (Bingham, Bacigalupi, & Johnson, 1997).

Urchins were maintained at the University of California Santa Barbara in flow‐through sea tables at ambient temperatures (12–15°C) and fed giant kelp (*Macrocystis pyrifera)* blades ad libidum prior to spawning. In order to follow the natural spawning season at each site and availability of gravid urchins, fertilization trials had to be run sequentially by site: February‐March (SB), April (BMR), and May (FC).

All fertilization trials took place in a temperature‐controlled aluminum block (~15°C) fitted for 25‐ml scintillation vials, for three pH treatments per unique male–female pair. Each male and female were used in only one set of experiments (i.e., males and females were not crossed with different individuals). Fertilization trials were replicated at the pair level to gain inferences on pair‐specific pH sensitivity, in sacrifice of technical replicates. Fertilization trials of two pairs were run per day. A total of 12 replicate fertilization trials were conducted on urchins collected from SB and FC. Only 10 replicate fertilization trials were conducted on those collected from BMR, as more urchins failed to spawn with induction.

Individuals were induced to spawn by injecting 0.5 mol/L KCl into the oral surface, at room temperature. Females were inverted on a beaker with ~15°C FSW to collect eggs. Immediately following spawning of each female, eggs were diluted to 0.44 eggs/μl (1,000 eggs per 2.3 ml) in treatment seawater, and allowed to acclimate for 10 min before fertilization at ~15°C. Eggs were checked for quality via shape, size, and color, and sperm was checked for motility, prior to fertilization. For each male, sperm was pipetted dry directly after extrusion from the gonopores, briefly centrifuged (to obtain near‐equal concentrations of concentrated sperm across males), and stored on ice while experimental vials were prepared. Just prior to use, concentrated sperm was diluted 1:10 in FSW from which three stock solutions were made in treatment water (1:100 final dilution). Sperm concentration of each stock solution was determined by preserving a subsample in 2% formaldehyde–seawater that was later scored on a hemocytometer and extrapolated for each serial dilution in the fertilization trial (Sewell et al., 2014).

The pH treatment order for preparation and fertilization was randomized for each fertilization trial. Serial sperm dilutions of 1:10 were set up by adding 2.3 ml of the stock solution to 20.7 ml treatment water in 25‐ml scintillation vials for a total of eight sperm concentrations (Levitan, Terhorst, & Fogarty, 2007; Reuter et al., 2011; Sewell et al., 2014). Vials were inverted between dilutions to ensure homogeneity. Eggs (1,000 eggs/2.3 ml) were added to the vials within 4.5 min of sperm activation in treatment water and gently inverted. Sperm–egg contact time was limited to 30 s and controlled by adding 1 ml 0.5 KCl to the vials and inverting to inhibit fertilization (Farley & Levitan, 2001). Embryos were subsequently allowed 2–4 hr for development at ambient temperatures in the sea table. The first 200 embryos encountered on a Sedgewick rafter slide were scored (two‐cell to eight‐cell stage). The following scoring metrics were used: normal fertilization (smooth raised fertilization membrane and equal cleavage if present), unfertilized (no fertilization membrane), tight fertilization membrane (Sewell et al., 2014; Tyler & Scheer, 1937), and abnormal development (normal fertilization envelope and unequal cleavage). At each sperm concentration, the proportion total eggs fertilized (TF), proportion of fertilized eggs that exhibit abnormal fertilization (AbnF), and proportion of total eggs with normal fertilization (NF) were calculated as follows, where the numerator is the response and denominator is the total count:

(1) TF=(#Tot.Eggs−#Unfert.)/#Tot.Eggs

(2) $$\begin{array}{cc} \text{AbnF}\;=\; & (\#\text{Tight Fert}.\text{Membr}.\;+\;\#\text{Abnorm}.\text{Dev}.)/\\ & \left(\#\text{Tot}.\text{Eggs}\;-\;\#\text{Unfert}.\right) \end{array}$$

(3) NF=TF∗(1−AbnF)

For AbnF, tight fertilization membrane and abnormal development were grouped, as both phenomena relate to fertilization and tight fertilization membrane can lead to abnormal development (Tyler & Scheer, 1937). Polyspermy is prevalent at high sperm concentrations and was the most likely cause for abnormal early cleavage observed in our data at high sperm concentrations (Franke, Babcock, & Styan, 2002; Levitan, 2004; Levitan & Ferrell, 2006; Levitan et al., 2007; Sewell et al., 2014; Styan, 1998). Cells exhibiting NF but no cellular division were scored as NF, due to the inability to distinguish between delayed cleavage and AbnD. This caveat may result in a slight underestimate of AbnF and overestimate of NF. Likewise, the occasional observation of unfertilized eggs found at high sperm concentrations may be polyspermic due to failure to raise the fertilization envelope, which is known to occur in *Mesocentrotus franciscanus* (Levitan et al., 2007).

### 2.4 Statistical approach

Two statistical approaches were used to evaluate how site and pH treatments impact fertilization dynamics and provide different forms of inference about the experimental data. For statistical simplicity, we first used generalized linear mixed effects models (GLMMs) to evaluate how pH treatment and site impact the empirical relationship between sperm concentration and (1) TF and (2) AbnF, in the absence of assumptions of underlying fertilization mechanisms. This approach maintains flexibility for describing statistical relationships without addressing mechanisms. We then employed a mechanistic model of fertilization dynamics (i.e., fertilization kinetics) to generate inference regarding which mechanistic processes (rate of sperm–egg interaction vs. polyspermy block rate) may be affected by pH treatments for each site. The latter approach addresses very specific hypotheses but requires more rigid constraints and assumptions. The model in this case does not allow for more complicated pH effects, such as impacts on postfertilization development, and will only reflect results that are consistent for both TF and AbnF under a single modeling framework. For both approaches, pH treatments were analyzed categorically as pH varied slightly for each fertilization trial (Table 1).

**Table 1:** Experimental conditions for *Strongylocentrotus purpuratus* fertilization trials by site (means ± *SD*,* N* = 12 for FC and SB, *N* = 10 for BMR). Categorical pH treatments were determined as the mean pH treatment across sites: pH_T_ 8.03, 7.87, 7.76, and 7.61. Ω_a_ is aragonite saturation state, A_T_ is total alkalinity

Parameter	pH treatment	FC	BMR	SB
Temperature (°C)	8.03	14.8 ± 0.2	14.3 ± 0.3	15 ± 0.5
	7.87	–	–	15 ± 0.4
	7.76	14.7 ± 0.2	14.4 ± 0.3	15 ± 0.4
	7.61	14.8 ± 0.3	14.4 ± 0.3	–
pH_T_	8.03	8.02 ± 0.03	8.06 ± 0.05	8.01 ± 0.01
	7.87	–	–	7.87 ± 0.01
	7.76	7.76 ± 0.02	7.75 ± 0.01	7.76 ± 0.02
	7.61	7.61 ± 0.01	7.60 ± 0.01	–
pCO_2_ (μatm)	8.03	428 ± 39	378 ± 46	435 ± 10
	7.87	–	–	623 ± 11
	7.76	828 ± 38	857 ± 18	832 ± 49
	7.61	1,213 ± 42	1,233 ± 38	–
Ω_a_	8.03	2.16 ± 0.15	2.33 ± 0.22	2.11 ± 0.04
	7.87	–	–	1.61 ± 0.04
	7.76	1.28 ± 0.05	1.22 ± 0.03	1.27 ± 0.06
	7.61	0.93 ± 0.03	0.90 ± 0.03	–
A_T_ (μmol/kg)	8.03	2,246 ± 5	2,239 ± 2	2,227 ± 3
	7.87	–	–	2,230 ± 6
	7.76	2,246 ± 6	2,240 ± 3	2,226 ± 6
	7.61	2,245 ± 5	2,244 ± 3	–
Salinity	8.03	33.0 ± 0.2	33.1 ± 0.1	33.0 ± 0.0
	7.87	–	–	33.0 ± 0.0
	7.76	33.0 ± 0.2	33.0 ± 0.1	33.0 ± 0.0
	7.61	33.0 ± 0.2	33.0 ± 0.1	–

#### 2.4.1 Linear statistical analysis (GLMMs)

For individuals collected from three sites exposed to different in situ pH variability regimes, we estimated the effect of site and pH on fertilization success, separately for TF and AbnF functions in order to isolate processes driving initial fertilization from those influencing abnormal fertilization and development of fertilized eggs (i.e., polyspermy). To assess the influence of site and pH treatment on TF and AbnF, we estimated models that considered logit‐scale probabilities as a function of sperm concentration using a third‐order polynomial of log_10_(sperm concentration). We allowed this relationship to vary by site, pH treatment, and the three‐way interaction (fixed effects of site, pH treatment, and sperm concentration) and also allowed the function to vary randomly by pair and by pH treatment within pair. We estimated models using GLMMs with a binomial likelihood estimated with the lme4 R package (Bates, Maechler, Bolker, & Walker, 2015). Because model residuals exhibited overdispersion (larger error variance than accounted for under a binomial distribution), we added an additional Gaussian noise term on the logit‐transformed probabilities as in Okamoto (2016). For each model, we tested whether each term was statistically significant using likelihood ratio tests. Main effects were tested in the absence of all interactions containing the focal effect, while two‐way interactions were tested in the absence of the three‐way interaction. These contrasts were chosen because (1) any nonsignificant interactions may reduce statistical power, (2) one or more significant interactions already suggest a significant effect, and (3) we examine interaction effects in detail using effect size metrics (described below). We restricted data used in analyses to sperm concentrations where either TF or AbnF was not uniformly 0 or 100% (1 < sperm/μl < 2 × 10^5^ for TF and 1 × 10^2^ < sperm/μl < 2 × 10^7^ for AbnF) to reduce underdispersion in that region. We estimated variance explained by individual fixed effects and random effects terms as in Johnson's extension (Johnson, 2014) to the method of Nakagawa and Schielzeth (2013) implemented with the MuMIn R package (Bartoń, 2016).

We used the resulting TF and AbnF models with full three‐way interactions to generate effect size metrics. Specifically, we calculated the sperm concentration required to achieve (1) 50% NF (S_NF50_), (2) optimal NF (S_OptNF_), and (3) 25% AbnF (S_AbnF25_). We generated expected NF function using TF and AbnF (Equation (3)). For AbnF, 25% was chosen as it is the approximate observed extent for most treatments so as not to extrapolate beyond observation boundaries. For each site × pH treatment combination within each metric, we used the model fixed effects estimates and covariance matrices to numerically solve for the sperm concentrations required to achieve those metrics. To calculate 95% confidence intervals for estimates and all pairwise contrasts within each site for each metric (i.e., three contrasts for each site and each metric comparing each treatment), we used a fully parametric bootstrap wherein we extracted the mean and variance–covariance matrix of the fixed effects parameters and generated 1,000 random samples from the resulting multivariate normal distribution to generate the confidence intervals and *p*‐values. We inferred homogeneous groups (within site per metric) using *p*‐values with Bonferroni corrections for three comparisons (α = 0.05/3 = 0.017). A global NF function was estimated by pooling site and treatments for use as a visual reference to compare fertilization curves in figures.

#### 2.4.2 Mechanistic fertilization dynamics model

We employed the fertilization dynamics model of Okamoto (2016) to evaluate whether any observed changes in the fertilization functions were consistent with (1) shifts in per capita sperm–egg interaction rates (driven by changes in sperm competency or a diversity of other potential processes) or (2) changes in polyspermy block rate (rate at which eggs become invulnerable to a second fertilizer following first fertilization). In brief, the model employs a series of differential equations describing the fertilization process and is the most recent formal extension of the model proposed by Vogel, Czihak, Chang, and Wolf (1982). Other model forms, such as that of Styan (1998) and Millar and Anderson (2003), provide similar results. The model considers the fraction of normal fertilization at time *t* as:

(4) $$E_{N}\left(t\right)=\frac{\delta }{E_{T}}\int_{0}^{t}\frac{\beta E_{T}S_{0}\gamma \left[e^{t(\beta E_{T}\;-\;\delta \;+\;r)}-1\right]}{\text{exp}\left[t\left(\beta E_{T}+r\right)-\frac{\beta S_{0}\gamma \left(1\;-\;e^{-t(\beta E_{T}\;+\;r)}\right)}{\beta E_{T}\;+\;r}\right]\left(\beta E_{T}-\delta +r\right)}dt$$

where *S*_0_ is the initial sperm concentration, *E*_T_ is the total egg concentration, *r* is the sperm viability decay rate, and key parameters are β (the rate of sperm–egg interactions per no., per second), γ (the dimensionless product of the fraction of sperm that interact with the egg that are acceptable and fertilizable fraction of the egg surface), and δ (the rate at which eggs fertilized by a single sperm induce a polyspermy block per second). For full model derivation and description, see Okamoto (2016). A similar model with measured egg diameter substituted into β (i.e., β = egg size × β*, where β* is the new estimated parameter), to ensure differences were not due to differences in egg diameter among treatments, yielded no qualitative difference in results (not shown).

We estimated the model parameters in a hierarchical Bayesian framework, where we simultaneously estimated a unique mean value (i.e., ${\overset{¯}{\beta }}_{P,T}$, $\delta_{P,T}$, ${\overset{¯}{\gamma }}_{P,T}$) for each site × pH treatment combination, as well as letting those parameters vary randomly for each pair; we modeled pair level β and δ values as a lognormal [i.e., β_*i,P,T*_ ~ lognormal (${\overset{¯}{\beta }}_{P,T}$, σ_β_) & δ_*i,P,T*_ ~ lognormal (${\overset{¯}{\delta }}_{P,T}$. σ_δ_)] and γ, as truncated normal [γ_*i*_ ~ normal (${\overset{¯}{\gamma }}_{P,T}$. σ_γ_), 0.0001 *<* γ_*i*_ < 0.15]. We utilized a beta‐binomial likelihood where the response variable is number of normal fertilized eggs given the total number of eggs and a dispersion parameter (λ) controls for over or under dispersion beyond the constraints of a standard binomial. We utilized vague priors and sampled the model posterior using Hamiltonian Monte Carlo via Stan (Gelman, Lee, & Guo, 2015) using RStan (Stan Development Team, 2016). There is no analytical solution to the integral in Equation (4), so we numerically integrated the function within each sampling iteration using Gauss–Legendre quadrature rules. We used a Bayesian framework for both computational convenience and to integrate over large uncertainty in γ and pair‐level variation in parameter estimates. There was insufficient information to reliably estimate egg selectivity/sperm viability (γ), and thus, all estimates are marginalized over the uncertainty in γ at all levels. Because we limited sperm–egg contact time to 30 s, the sperm decay rate (*r*) has no influence on parameter estimates (sperm decay over 30 s is negligible, Okamoto, 2016), so we use the value estimated in Okamoto (2016) of *r* = 0.0003 for *S. purpuratus* from Santa Barbara, California. See Table S1 for the full list of priors and table of model posteriors.

## 3 Results

### 3.1 Effect of pH on fertilization

Fertilization response to pH was assessed in urchins from three sites with radically different pH variability regimes: wide with frequent low pH exposure (FC), intermediate (BMR), and narrow with rare low pH exposures (SB). Where pH effects were significant, lower pH increased concentrations of sperm required to achieve a given fertilization rate (i.e., right shifting the curve). The data were analyzed via two approaches. First, the more flexible statistical approach of using GLMMs identified site‐specific differences in fertilization metrics wherein urchin fertilization sensitivity to low pH was detected for FC and SB when sperm concentrations were limiting or near optimal (i.e., not so abundant as to produce substantial abnormal fertilization or abnormal development). Under these conditions, SB urchins exhibited a pH sensitivity across a smaller pH range (pH_T_ 7.76–8.03) compared to FC urchins (pH_T_ 7.61–8.03). For urchins from BMR, pH effects were only detected at sperm concentrations great enough to produce abnormal fertilization and development (a trend that was observed for SB and statistically significant for FC). Second, the mechanistic models revealed that only experimental results from SB were consistent with a pH‐driven shift in per capita rate of interaction among sperm and eggs (rate at which sperm collide with eggs).

### 3.2 Linear statistical analysis (GLMMs)

**Figure 3:**
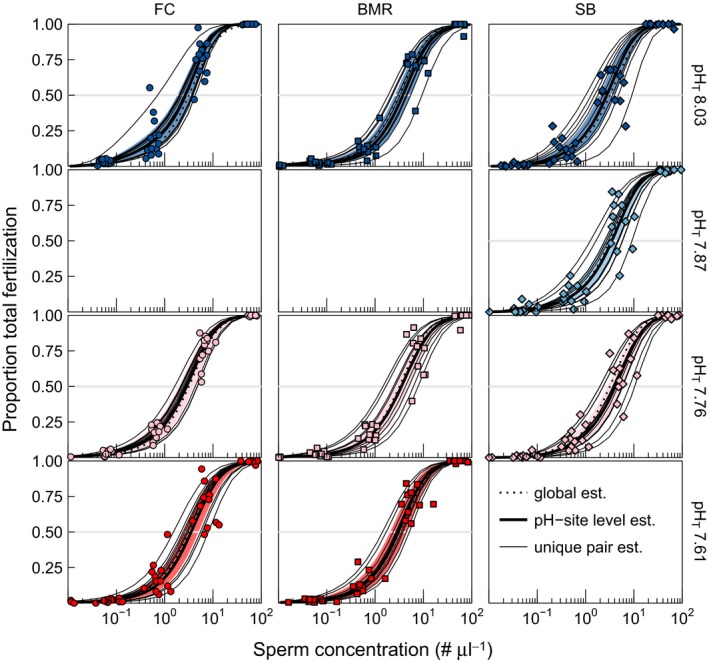
Proportion total fertilization over a range of sperm concentrations for *Strongylocentrotus purpuratus* from FC, BMR, and SB by pH treatment (colors are the same as in Figure 2b). The dotted line represents the global mean estimate. Solid, thick lines represent the site–pH treatment level estimates with the 95% confidence interval as the colored band. Thin, solid lines represent unique pair estimates (i.e., random effects) for each site–pH treatment. The horizontal line denotes 50% total fertilization. Only three pH treatments were tested per site

**Figure 4:**
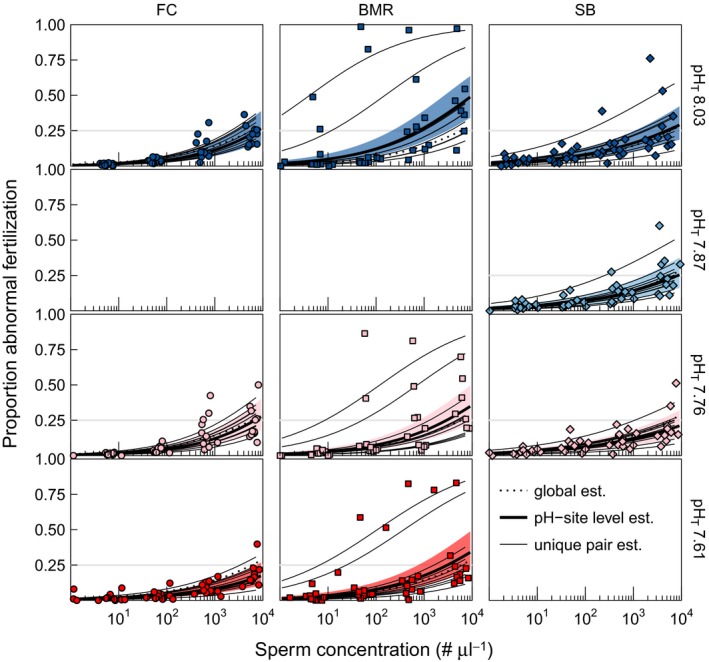
Proportion abnormal fertilization over a range of sperm concentrations for *Strongylocentrotus purpuratus* from FC, BMR, and SB by pH treatment (colors are the same as in Figure 2b). The dotted line represents the global mean estimate. Solid, thick lines represent the site–pH treatment level estimates with the 95% confidence interval as the colored band. Thin, solid lines represent unique pair estimates (i.e., random effects) for each site–pH treatment. The horizontal line denotes 25% abnormal fertilization. Only three pH treatments were tested per site

Site and pH treatment both exhibited a subtle influence on TF and AbnF. Sperm concentration was by far the greatest determinant of both TF and AbnF in our experiment. In addition, the mean (fixed) effect of sperm concentration alone explained 78% and 20% of variance in TF and AbnF, respectively. The combined addition of site and pH treatment fixed effects yielded an increase in only 1.7% and 2.6% of variance explained for TF and AbnF, respectively. Site and pH treatment accounted for a small, but statistically significant (Table 2), portion of the variation in TF and AbnF functions. The mean effect of pH on TF differed by site, as indicated by a three‐way sperm × site × pH treatment interaction (*p *<* *.05). In contrast, the AbnF function was impacted by sperm concentration, site × sperm interaction, and an overall pH effect. In addition, among‐pair variability in the response accounted for 2.4% and 12% of error variance, with the remaining 18% and 65% of error variance due to within pair variability (i.e., unexplained noise), in TF and AbnF, respectively. Put simply, most of the variation in AbnF was unexplained, while variation in TF was largely explained by sperm concentration alone; however, site and pH still had significant effects despite explaining much smaller percentages of total variation. Figure 2b visually illustrates the aggregate variation about the mean response of fertilization to sperm concentration, while TF and AbnF functions are shown in Figures 3 and 4 along with the estimated global fertilization function (pooled site and pH treatments) for comparison (see Supporting Information for individual fertilization functions).

**Table 2:** Likelihood ratio tests for generalized linear mixed models of total fertilization proportion and abnormal fertilization proportion. Main effects were tested in the absence of interactions involving the focal effect, and two‐way interactions were tested in the absence of the three‐way interaction (see the Methods section)

Source	Total fertilization			Abnormal fertilization		
	χ^2^	*df*	*p*	χ^2^	*df*	*p*
Sperm Conc.	1258.10	3	<.001	490.71	1	<.001
Site	1.45	2	.485	5.16	2	.076
pH treatment	5.81	3	.121	8.98	3	.030
Site × Sperm	27.24	6	<.001	14.41	2	<.001
pH × Sperm	8.56	9	.479	3.22	3	.360
Site × pH	17.27	3	<.001	5.24	3	.155
Site × pH × Sperm	23.05	9	<.01	1.08	3	.783

**Figure 5:**
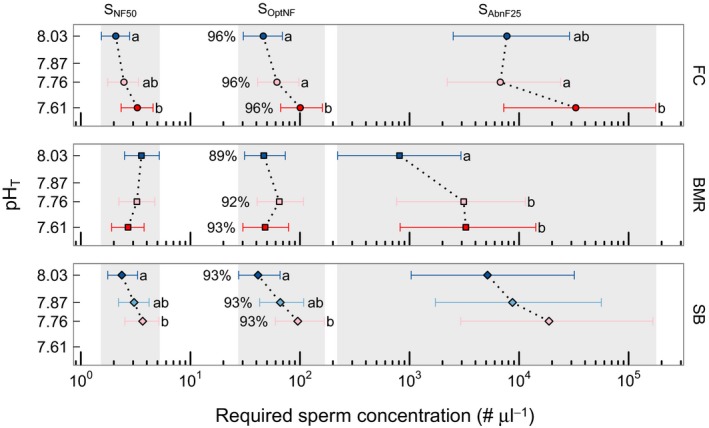
Estimated sperm concentrations required to reach 50% normal fertilization (S_NF__50_), optimal normal fertilization (S_O__pt_ _NF_), and 25% abnormal fertilization (S_A__bnF25_) under different pH treatments (*y*‐axis), for *Strongylocentrotus purpuratus* from FC, BMR, and SB. Points within a population‐metric combination that do not share a common letter within a metric are significantly different (α = 0.017, following a Bonferroni correction for three comparisons under a parametric bootstrap). Those with no letters indicate no significant differences among treatments (within site, per metric). Percentages are the estimated peak levels of normal fertilization. Error bars are 95% confidence intervals estimated via parametric bootstrap. Colors are the same as in Figure 2b

To quantify the interactive effect of site and pH treatment, pairwise comparisons of fertilization metrics (S_NF50_, S_OptNF_, and S_AbnF25_) were calculated, revealing site‐specific pH sensitivities in urchin fertilization (Figure 5). While estimated peak levels of NF were comparable across treatments, both FC and SB urchins exhibited a trend (significant or nonsignificant) in right shifting fertilization curves with decreasing pH (i.e., more sperm required to achieve the same levels of normal, optimal, and abnormal fertilization indicated by S_NF50_, S_OptNF_, and S_AbnF25_, respectively). Changes in sperm concentration with fertilization metrics differed significantly for S_NF50_ and S_OptNF_, at both FC and SB (α = 0.017, following Bonferroni corrections for three comparisons, Table S2). For SB, this meant that slightly higher sperm concentrations were required to reach S_NF50_ and S_OptNF_ at pH_T_ 7.76, compared to pH_T_ 8.03. For the FC, the same effect was observed; however, the lowest pH that differed significantly from control pH_T_ 8.03 was pH_T_ 7.61, and not pH_T_ 7.76 as was the case for SB. Thus, while both SB and FC exhibited sensitivity in S_NF50_ and S_OptNF_ to decreasing pH, the pH range over which this sensitivity was observed was smaller at SB (pH_T_ 8.03 vs. 7.76) compared to FC (pH_T_ 8.03 vs. 7.61). In contrast, urchins from BMR exhibited no statistically significant pH sensitivity for either S_NF50_ or S_OptNF_. BMR urchins did exhibit a right shift in S_AbnF25_ at pH_T_ 7.61 compared to pH_T_ 8.03 (Figure 5), as did FC urchins but from pH_T_ 7.76 to pH_T_ 7.61. Two urchin pairs from BMR exhibited unusually high percentage of AbnD (Figure 4). Excluding these two pairs from analyses neither changed observed patterns for fertilization metrics nor the conclusions (see Supporting Information).

### 3.3 Mechanistic fertilization dynamics model

**Figure 6:**
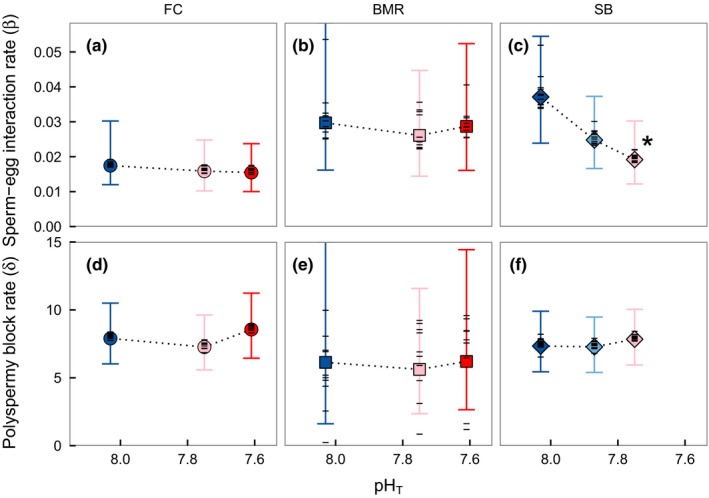
Parameter estimates for the instantaneous per capita sperm–egg interaction rate (per no., per second, a‐c) and polyspermy block rate (per second, d‐f) by pH treatment, for *Strongylocentrotus purpuratus* from FC (a, d), BMR (b, e), and SB (c, f), using the mechanistic model of Okamoto (2016). Large symbols represent among‐pair means with 95% posterior credibility intervals (error bars) and horizontal dashes are the pair‐specific mean estimates. Asterisk denotes mean estimates with >95% posterior probability of a decline compared to the ambient (pH_T_ = 8.03) treatment. Colors are the same as in Figure 2b

To identify the mechanistic underpinnings of the observed site‐specific pH sensitivities in fertilization success, we estimated the instantaneous, per capita sperm–egg interaction rate (β) and polyspermy block rate (δ) for each site × pH treatment (Figure 6). For sperm–egg interaction rate, we only detected a meaningful change across pH treatments in urchins from SB, which was the site with the narrowest pH variability regime. For SB, sperm–egg interaction rate (β) declined by an estimated 46% from pH_T_ 8.03 to pH_T_ 7.76 (upper and lower 95% credible set = 14%–69%, Table S1), with 98% posterior probability of a decline at this treatment. In contrast, we detected no meaningful change in β for either FC or BMR (Figure 6, Table S1). No other site–treatment combinations had >95% probability of decline over the control (pH_T_ 8.03) treatment. For polyspermy block rates, we detected no meaningful effect of pH treatment for any site (Figure 6). This indicates that pH sensitivity of *S. purpuratus* fertilization in this study was related to the pH sensitivity of sperm (and not eggs), which is also shown by the subtle right shift in the fertilization function across sperm concentration (i.e., more sperm required to reach fertilization metrics, Figure 5).

## 4 Discussion

We investigated the pH sensitivity of fertilization in sea urchins from different coastal ocean pH variability regimes. For *S. purpuratus* collected from three sites, pH sensitivity of fertilization was greatest in urchins from the site exposed to the narrowest pH variability regime (SB). Here, the negative pH effect was observed at pH_T_ 7.76 via a right shift in the fertilization function. For urchins from sites with more frequent low pH exposures (FC, BMR), low pH tolerance extended by ≥0.11 units pH_T_. In other words, urchins from sites with frequent low pH exposure required much lower pH treatments to significantly alter fertilization rates. These results are consistent with the climate variability hypothesis and emphasize the need to include spatial and temporal environmental variability in studies of species responses to environmental change (Figure 1).

Across all sites, high mean fertilization success (89%–96%, Figure 5) was achieved at pH_T_ ≥ 7.6 as previously shown for *S. purpuratus* (Frieder, 2014) and other urchin species (Byrne, 2011; Moulin et al., 2011; Reuter et al., 2011). The effect of site and pH treatment on fertilization was small compared to that of sperm concentration or unexplained variation. The following discussion is devoted to the significance of these results in terms of assessing (1) how pH variability might shape adaptive capacity of *S. purpuratus* to ocean acidification, and (2) the ecological implications of the observed pH sensitivity, as ocean acidification might exert only a subtle influence on fertilization dynamics.

### 4.1 Adaptive capacity

The extended low pH tolerance in fertilization dynamics of urchins from FC and BMR compared to SB could occur as a transgenerational response to in situ low pH exposures (Ross et al., 2016) or stem from natural selection (e.g., postsettlement selection, local adaptation; Kelly et al., 2013; Pespeni, Chan, et al., 2013). Our results are consistent with recent studies showing local adaptation of *S. purpuratus* to pH (Evans, Pespeni, Hofmann, Palumbi, & Sanford, 2017; Kelly et al., 2013; Pespeni, Chan, et al., 2013; Pespeni, Sanford, et al., 2013). Kelly et al. (2013) found that *S. purpuratus* larvae grew larger at pH_T_ 7.60 when the sires originated from a site exposed to stronger upwelling events and lower pH (Van Damme State Park, ~140 km north of BMR) than when sires originated from a site with narrow pH variability (SB). The potential for local adaptation of *S. purpuratus* in response to pH is further supported by observations of changes in allelic frequencies associated with low‐pH adapted alleles of adults across the CCLME (Pespeni, Chan, et al., 2013).

Our results, taken with the context above, indicate that pH tolerance in *S. purpuratus* is likely spatially optimized to current exposures because sensitivities were detected at pH levels that locally occur more rarely (Figure 1). Such spatial fine‐tuning suggests that *S. purpuratus* has at least some adaptive capacity (i.e., ability to physiologically adjust via transgenerational plasticity or to evolve) to deal with ocean acidification over temporal scales. In the laboratory, other urchin species have exhibited beneficial transgenerational responses to low pH exposures, when adults were exposed to low pH over a full annual cycle of reproductive conditioning (Dupont, Dorey, Stumpp, Melzner, & Thorndyke, 2013; Suckling et al., 2015).

The cellular and physiological mechanisms that regulate the pH sensitivity of key functional traits, such as fertilization, shape the basis for assessing adaptive capacity but remain underdescribed. Using related mechanistic models, studies on other genera have inferred an impact of pH on polyspermy block rate (Sewell et al., 2014) and sperm–egg interaction rates (Reuter et al., 2011), suggesting potential species‐specific sensitivities (Frieder, 2014). For *S. purpuratus*, we did not detect pH effects on block rates. For urchins from SB, however, sperm–egg interaction rates declined with pH, which could be driven by reduced sperm competency. Sperm motility is considered an important factor contributing to observations of reduced urchin fertilization success in laboratory pH experiments (Bögner, 2016; Havenhand et al., 2008). Sperm motility is activated by a pH‐dependent, ATP‐hydrolyzing, enzyme complex, axonemal dynein ATPase. Activation of dynein ATPase depends on H^+^ extrusion by Na^+^‐H^+^ exchangers that increase intracellular pH (pH_i_) once the sperm is released into seawater (Bögner, 2016). As such, dynein ATPase activity increases linearly with pH_i_ from 7.4 to 8.0 and is directly related to sperm motility (Trimmer & Vacquier, 1986; but see Caldwell et al., 2011). Seawater pH may influence sperm motility by modulating pH_i_ through direct intracellular acidification via CO_2_ diffusion across the cell membrane (i.e., acidosis) or alteration of the effectiveness of transmembrane proteins that control pH_i_. Given this mechanism, adult pH exposure could enhance gamete performance via changes in gametic control of pH_i_. For example, frequent low pH exposure of adults may impact the number or efficiency of transmembrane proteins available for pH_i_ homeostatis in sperm, as discussed by Moulin et al. (2011), potentially through epigenetic changes or selection over time.

Similarly for females, adaptive control on egg pH_i_ could alleviate intracellular acidosis that might underpin the delayed polyspermy block at low pH observed in *M. franciscanus* and *Sterechinus neumayeri* (Reuter et al., 2011; Sewell et al., 2014). We did not observe this effect in *S. purpuratus* (Figure 6). Instead, at the site where pH treatment effects were strongest, the overall sperm–egg interaction rate was impacted with no detectable impact on polyspermy block dynamics (Figure 6). This result may indicate enhanced pH sensitivity of sperm (i.e., enzymes that control sperm motility, viability, binding, and gamete recognition) or factors that contribute to interaction rates (i.e., attractive properties of eggs). Selection effects on males may therefore be the source of varying local pH sensitivities in *S. purpuratus* (Kelly et al., 2013).

The data from BMR, the site of intermediate pH variability but of urchins with greatest pH tolerance, illustrate that pH sensitivity is not simply a function of frequency of low pH events ≤7.80. Local stress events could influence fertilization dynamics. For example, months prior to the urchin collections at BMR, there had been a rapid mortality event of intertidal *S. purpuratus* (E. Sanford, pers. comm.). This could have influenced surviving individuals differently (e.g., maturation or quality of the gonads) and the BMR urchins that did survive to spawn may have, inadvertently, been more tolerant of environmental stressors than what is representative of the BMR population in general. Urchin densities worldwide are dynamic in space and time with occasional boom and bust cycles (Filbee‐Dexter & Scheibling, 2014) and it could be that such short‐term perturbations have strong effects in terms of site tolerances at a given location and moment in time. Regardless of the cause, the BMR results contribute valuable information regarding the presence of pH‐resistant pairs within a population.

### 4.2 Ecological implications

From an ecological perspective, pH has a much smaller impact on fertilization dynamics than sperm availability, pair compatibility, or other unidentified processes. First, sperm availability (accounting for 78% of variation in TF) is an obvious determinant of fertilization success. In our experiment, the statistically significant differences in fertilization metrics (e.g., S_OptNF_) occurred within or across one order of magnitude in sperm concentration. However, urchin fertilization rates during broadcast spawning events can vary dramatically in the wild and gradients in sperm concentration can span multiple orders of magnitude (Franke et al., 2002; Levitan, 2002, 2004). Sperm concentration, sperm quality, and rates of sperm–egg encounters are dominant factors determining fertilization success (Levitan, Sewell, & Chia, 1991). In the field, these factors are influenced by urchin density of the spawning population (Gaudette, Wahle, & Himmelman, 2006; Levitan, 2002; Levitan et al., 1991; Wahle & Peckham, 1999), local sex ratios (Levitan, 2004), rates of gamete advection (Lauzon‐Guay, Scheibling, & Barbeau, 2006; Levitan, 2005), and hydrodynamic mixing (Crimaldi, 2012).

Second, we observed some among‐pair and substantial unexplained variation in fertilization responses. Less than 3% of variation in *S. purpuratus* fertilization curves was explained by site and pH treatment, whereas pair effects accounted for up to 12% of variation in AbnF. Pair‐specific compatibility varies dramatically in *S. purpuratus* (Levitan & Stapper, 2010; Stapper, Beerli, & Levitan, 2015) as well as in *M. franciscanus* (Levitan, 2012; Levitan & Ferrell, 2006). Even so, the competitive advantage of specific males during a mass spawning event could be pH dependent (Campbell et al., 2016). Unexplained variation could stem from variation in pH dependencies of processes involved to create a fertilization event other than those measured in this study (e.g., proteins involved in gamete fusion and others discussed by Campbell et al., 2016).

Pair‐dependent pH sensitivities have been observed in fertilization dynamics of other urchins, *Heliocidaris erythrogramma* (Schlegel et al., 2012) and *S. neumayeri* (Sewell et al., 2014). Our data are consistent with the hypothesis that environmental variability may act on pair‐specific sensitivities, thereby increasing the pH tolerance window of local urchin aggregations (Figure 1). Maintaining diverse variation in among‐pair compatibility and pH sensitivity (e.g., large, genetically diverse populations) may facilitate the adaptive response to environmental change, despite the fact that among‐pair compatibility may be a stronger factor driving fertilization success than pH. However, as fertilization has direct fitness costs, it remains a critical step in population persistence. Small, but widespread shifts in fertilization function due to ocean acidification could still have an important impact, but this process will not likely be a bottleneck for marine invertebrates in the future (Byrne, 2011).

As global ocean change progresses, assessing the adaptive capacity of marine species is of increasing interest to researchers and coastal ocean management groups (Chan et al., 2016). Protecting breeding populations diverse in the pH sensitivities of their functional traits may become an important management approach, especially if such populations are sources to others (Sanford & Kelly, 2011). In addition to urchins (Ross et al., 2016), other marine species show beneficial transgenerational effects from adult pH exposures (oysters, Parker, O'connor, Raftos, Pörtner, & Ross, 2015; Parker et al., 2012; mussels, Fitzer, Cusack, Phoenix, & Kamenos, 2014; fish, Miller, Watson, Donelson, Mccormick, & Munday, 2012; Munday, 2014; Murray, Malvezzi, Gobler, & Baumann, 2014; copepods, Pedersen et al., 2014; Thor & Dupont, 2015; and corals, Putnam & Gates, 2015). Thus, identifying habitats with unique oceanographic features that select for pH‐resistant traits among multiple species will be important for conservation efforts (e.g., management of other local stressors) and tracking ecological change. Specifically, it may be that such regions are “nurseries” for genetic diversity. Hot spots of low pH with wide pH variability may, however, at the same time be more sensitive to ocean change compared to sites with narrow pH variability regimes. The interplay of environmental variability and subsequent ecological interactions (Kroeker et al., 2016) presents a new research frontier for understanding the effects of environmental variability and anthropogenic activities on marine ecosystems.

## Conflict of Interest

None declared.
