# Supplementary material for: Sensitivity of sea urchin fertilization to pH varies across a natural pH mosaic
Source: Ecol Evol. 2017 Feb 12;7(6):1737–50. doi: 10.1002/ece3.2776 (PMC5355180; doi:10.1002/ece3.2776)
Supplement: Supplementary file 1 [file ECE3-7-1737-s001.docx]

**Supporting Information**. This document contains the results tables for the mechanistic model and fertilization metrics analysis, discussion on outliers, and figures of individual fertilization curves.

**Table S1.** Results table for the mechanistic model (Okamoto, 2016) of fertilization.

| **Parameter** | **Description** | **Site** | **pH_T_** | **Value** | **Credible Set** | | | **Prior** |
| --- | --- | --- | --- | --- | --- | --- | --- | --- |
| 𝜷_𝜇_ | Sperm-egg collision rate (sperm^-1^ egg^-1^ second ^-1^) | FC | 8.03 | 0.0175 | 0.012 | : | 0.0583 | Uniform (0-0.1) |
|  |  |  | 7.76 | 0.0159 | 0.0102 | : | 0.0447 |  |
|  |  |  | 7.61 | 0.0155 | 0.01 | : | 0.0524 |  |
|  |  | BMR | 8.03 | 0.0297 | 0.0162 | : | 0.0302 |  |
|  |  |  | 7.76 | 0.0261 | 0.0144 | : | 0.0248 |  |
|  |  |  | 7.61 | 0.0287 | 0.0161 | : | 0.0237 |  |
|  |  | SB | 8.03 | 0.0371 | 0.0239 | : | 0.0545 |  |
|  |  |  | 7.87 | 0.0248 | 0.0166 | : | 0.0373 |  |
|  |  |  | 7.76 | 0.0192 | 0.0122 | : | 0.0302 |  |
| 𝛿_𝜇_ | Polyspermy block rate (second^-1^) | FC | 8.03 | 7.9 | 6.02 | : | 10.5 | Uniform (0-30) |
|  |  |  | 7.76 | 7.26 | 5.58 | : | 9.62 |  |
|  |  |  | 7.61 | 8.56 | 6.44 | : | 11.23 |  |
|  |  | BMR | 8.03 | 6.14 | 1.62 | : | 25.65 |  |
|  |  |  | 7.76 | 5.62 | 2.35 | : | 11.57 |  |
|  |  |  | 7.61 | 6.2 | 2.65 | : | 14.43 |  |
|  |  | SB | 8.03 | 7.34 | 5.44 | : | 9.9 |  |
|  |  |  | 7.87 | 7.28 | 5.39 | : | 9.47 |  |
|  |  |  | 7.76 | 7.84 | 5.94 | : | 10.04 |  |
| 𝛾_𝜇_ | Egg selectivity/ Sperm compatibility | FC | 8.03 | 0.1091 | 0.0252 | : | 0.1483 | Uniform (0-.15) |
|  |  |  | 7.76 | 0.0975 | 0.0054 | : | 0.1478 |  |
|  |  |  | 7.61 | 0.0749 | 0.0039 | : | 0.1474 |  |
|  |  | BMR | 8.03 | 0.0729 | 0.0019 | : | 0.1479 |  |
|  |  |  | 7.76 | 0.0748 | 0.0034 | : | 0.1454 |  |
|  |  |  | 7.61 | 0.0788 | 0.0046 | : | 0.1451 |  |
|  |  | SB | 8.03 | 0.0814 | 0.0061 | : | 0.1461 |  |
|  |  |  | 7.87 | 0.0765 | 0.0058 | : | 0.1453 |  |
|  |  |  | 7.76 | 0.0807 | 0.0047 | : | 0.1482 |  |
| 𝝺 | total count (dispersion ^-1^) |  |  | 6.98 | 6.39 | : | 7.63 | Pareto (1,1.5) |
| 𝜎_β_ | among pair sd in 𝜷 (lognormal) |  |  | 0.3 | 0.02 | : | 0.95 | Uniform (0.0001-4) |
| 𝜎_𝛿_ | among pair sd in 𝛿 (lognormal) |  |  | 0.54 | 0.02 | : | 2.14 |  |
| 𝜎_𝛾_ | among pair sd in 𝛾 (trunc. normal) |  |  | 1.56 | 0.01 | : | 3.88 |  |

| **Table S2.** Table of parametric bootstrap *p*-values for comparisons (pH:site) of fertilization metric estimates (S_NF50_, S_OptNF_, and S_AbnF25_). *P*-values represent proportion of bootstrap samples where row entry > column entry (i.e., *p* = α or 1-α means row treatment is significantly different than column treatment, with the former representing row < column and the latter row > column). We set α = 0.017 (=0.05/3, for Bonferroni corrections for 3 comparisons per site) to compare treatment effects within sites (shaded, represented in Fig. 5, *denotes significance). | | | | | | | | | |
| --- | --- | --- | --- | --- | --- | --- | --- | --- | --- |
| ***Metric*** |  | **8.03:FC** | **7.76:FC** | **7.61:FC** | **8.03:BMR** | **7.76:BMR** | **7.61:BMR** | **8.03:SB** | **7.87:SB** |
| **S_NF50_** | *7.76:FC* | 0.111 |  |  |  |  |  |  |  |
|  | *7.61:FC* | 0.001* | 0.03 |  |  |  |  |  |  |
|  | *8.03:BMR* | 0.01 | 0.931 | 0.614 |  |  |  |  |  |
|  | *7.76:BMR* | 0.032 | 0.147 | 0.481 | 0.712 |  |  |  |  |
|  | *7.61:BMR* | 0.152 | 0.358 | 0.793 | 0.953 | 0.846 |  |  |  |
|  | *8.03:SB* | 0.722 | 0.443 | 0.078 | 0.049 | 0.113 | 0.286 |  |  |
|  | *7.87:SB* | 0.039 | 0.817 | 0.39 | 0.745 | 0.405 | 0.696 | 0.043 |  |
|  | *7.76:SB* | 0.005 | 0.956 | 0.683 | 0.456 | 0.695 | 0.891 | 0.005* | 0.127 |
|  |  | **8.03:FC** | **7.76:FC** | **7.61:FC** | **8.03:BMR** | **7.76:BMR** | **7.61:BMR** | **8.03:SB** | **7.87:SB** |
| **S_OptNF_** | *7.76:FC* | 0.104 |  |  |  |  |  |  |  |
|  | *7.61:FC* | 0.001* | 0.009* |  |  |  |  |  |  |
|  | *8.03:BMR* | 0.488 | 0.184 | 0.008 |  |  |  |  |  |
|  | *7.76:BMR* | 0.164 | 0.44 | 0.085 | 0.075 |  |  |  |  |
|  | *7.61:BMR* | 0.454 | 0.783 | 0.984 | 0.465 | 0.893 |  |  |  |
|  | *8.03:SB* | 0.371 | 0.099 | 0.003 | 0.362 | 0.098 | 0.32 |  |  |
|  | *7.87:SB* | 0.139 | 0.585 | 0.092 | 0.143 | 0.505 | 0.837 | 0.029 |  |
|  | *7.76:SB* | 0.013 | 0.912 | 0.429 | 0.017 | 0.858 | 0.981 | <0.001* | 0.081 |
|  |  | **8.03:FC** | **7.76:FC** | **7.61:FC** | **8.03:BMR** | **7.76:BMR** | **7.61:BMR** | **8.03:SB** | **7.87:SB** |
| **S_AbnF25_** | *7.76:FC* | 0.605 |  |  |  |  |  |  |  |
|  | *7.61:FC* | 0.019 | 0.010* |  |  |  |  |  |  |
|  | *8.03:BMR* | 0.991 | 0.003 | <0.001 |  |  |  |  |  |
|  | *7.76:BMR* | 0.855 | 0.805 | 0.009 | 0.005* |  |  |  |  |
|  | *7.61:BMR* | 0.824 | 0.78 | 0.988 | 0.01* | 0.457 |  |  |  |
|  | *8.03:SB* | 0.353 | 0.39 | 0.076 | 0.963 | 0.675 | 0.653 |  |  |
|  | *7.87:SB* | 0.469 | 0.583 | 0.14 | 0.012 | 0.831 | 0.804 | 0.261 |  |
|  | *7.76:SB* | 0.233 | 0.799 | 0.336 | 0.003 | 0.942 | 0.924 | 0.055 | 0.189 |

**Figure S1.** Normal fertilization functions by pH treatment for individual urchin pairs from FC, BMR, and SB. Thin dotted lines represent the global curve (pooled site and pH treatment).

**Figure S2.** Total fertilization functions by pH treatment for individual urchin pairs from FC, BMR, and SB. Thin dotted lines represent the global curve (pooled site and pH treatment).

**Figure S3.** Abnormal fertilization functions by pH treatment for individual urchin pairs from FC, BMR, and SB. Thin dotted lines represent the global curve (pooled site and pH treatment).

**Discussion on outliers –** Two urchin pairs from BMR (pair 2 and 3) exhibited unusually high percentage of AbnF (Fig. 4, Fig. S3). Excluding these two pairs from analyses did not change observed patterns for fertilization metrics but resulted in significant differences for FC: S_NF50_ at pH_T_ 7.76 became statistically different from pH_T_ 7.61, and S_AbnF25_ at pH_T_ 8.03 became statistically different from pH_T_ 7.61. This was due to an overall decrease in the pooled estimate of among-pair variability in pH treatment response for AbnF (analysis not shown). In the presence of outliers, both comparisons neared statistical significance (*p* = 0.030 and 0.019 for S_NF50_ and S_AbnF25_, respectively with α = 0.017, following adjustment for multiple comparisons, Table S2). Otherwise, results are qualitatively identical including Likelihood ratio tests and mechanistic model results.

**References**

Okamoto DK (2016) Competition among eggs shifts to cooperation along a sperm supply gradient in an external fertilizer. The American Naturalist*,* **187**, E129-E142
